# Reverse Abdominoplasty for Reconstruction Following Oncologic Resection of Extensive Breast Disease

**DOI:** 10.7759/cureus.28664

**Published:** 2022-09-01

**Authors:** Matthew Culbert, Leslie Shock, Michela M Fabricius, Nicole Nelson

**Affiliations:** 1 Radiation Oncology, University of Florida College of Medicine, Gainesville, USA; 2 Plastic Surgery, University of Missouri School of Medicine, Columbia, USA; 3 General Surgery, University of Missouri School of Medicine, Columbia, USA

**Keywords:** general surgery, ductal carcinoma in-situ, paget’s disease, chest wall resection, breast cancer management, reverse abdominoplasty flap

## Abstract

We present two cases of patients with extensive breast disease who underwent a reverse abdominoplasty for closure following resection: one of Paget’s disease extending beyond the breast borders and another of a locally recurrent triple-negative invasive ductal carcinoma following mastectomy in a patient who previously had an ipsilateral thoracotomy. The reverse abdominoplasty flap is a reconstructive option not readily considered for closure following mastectomy. However, we believe that the reverse abdominoplasty flap should be considered when evaluating patients for anterior chest wall reconstruction because it is a simple and versatile coverage option.

## Introduction

The reverse abdominoplasty flap was first described by Rebello and Franco in the 1970s [1]. It is an advancement flap created through a submammary incision and is traditionally used for the correction of excess supraumbilical skin [1]. Applications of this technique have expanded to include contouring surgery with plastic surgery and more recently, it has been employed in a few reconstructive procedures following oncologic resection [2-5].

A reverse abdominoplasty as a standalone method of closure following oncologic resection is uncommon. This is largely due to the extensive options available for closure of soft tissue defects, such as the pectoralis muscle flap, the omental flap, the latissimus dorsi flap, the transverse rectus abdominis flap, free flaps, and skin grafts, among others [6]. Vascular supply, quick coverage, and the extensive nature of the disease are all options that weigh into the decision of the type of flap used.

We present two cases of the application of the reverse abdominoplasty flap for anterior chest wall soft tissue reconstruction following unilateral mastectomy and wide local excisions. The first case involves a patient with extensive Paget’s disease of the breast, and the second case involves a patient with a large area of locally recurrent disease following ipsilateral thoracotomy and mastectomy. Neither patient had received radiation before the intervention. These cases were previously presented as a podium presentation at the Missouri Chapter of the American College of Surgeons on April 30, 2022.

## Case presentation

Case 1

**Figure 1:**
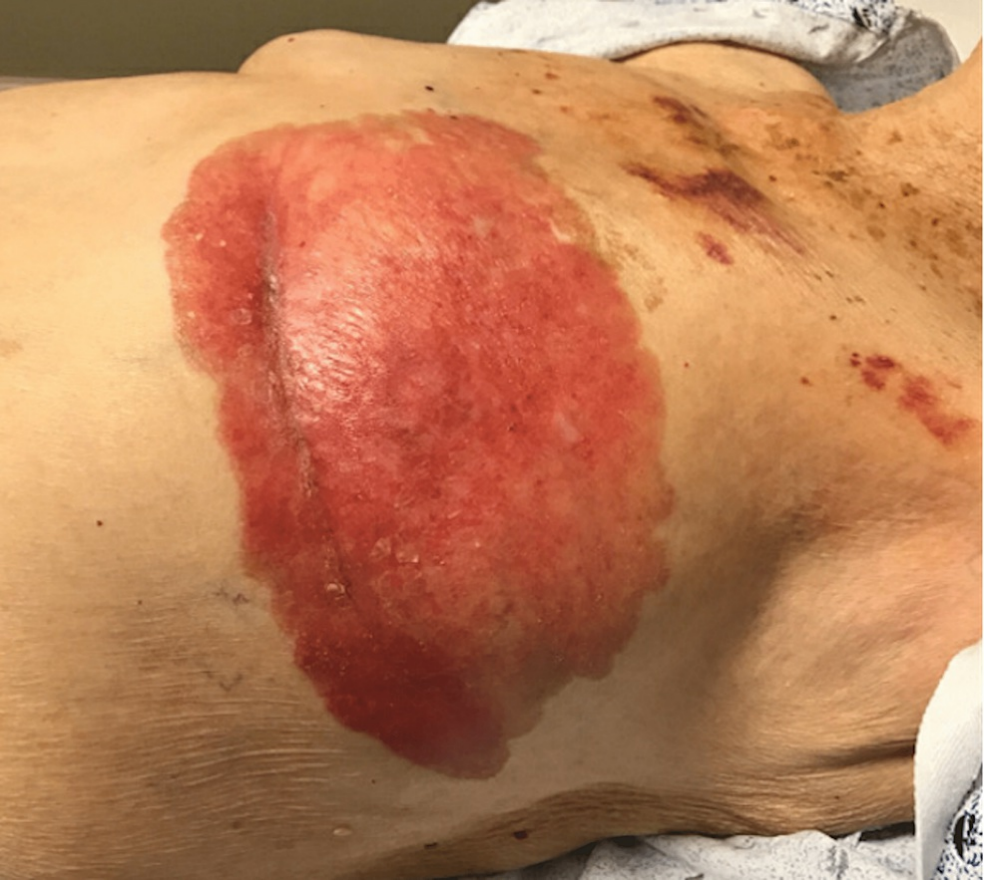
Case 1 - Initial presentation Extensive (22 x 12 cm) Paget’s disease of the breast extending beyond the breast borders with underlying ductal carcinoma in situ. There is the obliteration of the nipple with disease extending 2 cm below the inframammary fold and crossing the midline.

An 86-year-old female presented with a five-year history of eczematoid skin changes over her left breast extending over the left chest wall (Figure 1). Pathologic investigations revealed Paget’s disease extending beyond the borders of her breast with associated underlying ductal carcinoma in situ of the breast. Imaging did not reveal any underlying abnormality of the breast parenchyma or nearby structures. Closure options were limited due to the potential size of the defect as well as potential surgical and donor site morbidity.

**Figure 2:**
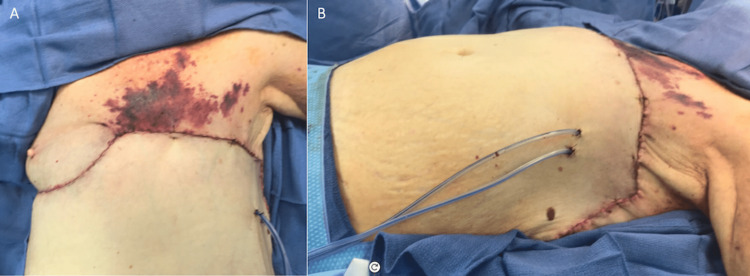
Case 1 - Excision & closure (A) Complete closure of the anterior chest wall defect (16 x 36 cm) following left mastectomy with an axillary sentinel lymph node biopsy and a wide local excision. The inferior margin constituted the superior border of the reverse abdominoplasty flap. (B) Drains were placed with multi-layered closure.

Therefore, a mastectomy with the resection of the involved skin with a reverse abdominoplasty flap for closure following resection was advised. The patient declined breast reconstruction due to not wanting to undergo numerous procedures. On the day of the surgery, the total excisional area measured 16 x 36 cm and there was no tension on closure (Figure 2). Final pathology revealed Paget’s disease of the breast with underlying ductal carcinoma in situ and some lobular carcinoma in situ. The margins were clean.

**Figure 3:**
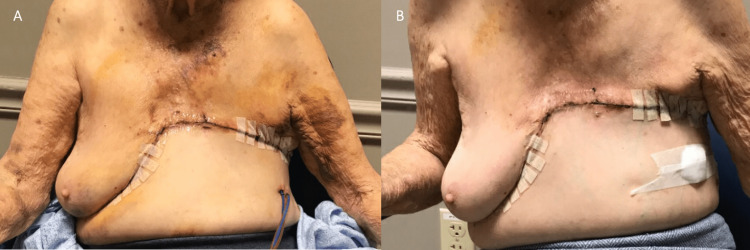
Case 1 - Follow up (A) One-week postoperative follow-up with drains still in place and mild ecchymosis on the skin. (B) Three-week postoperative follow-up with the removal of drains.

The patient had no immediate postoperative complications or complications during subsequent follow-ups (Figure 3). She had no evidence of recurrence six months later (six-month images are not available) and demonstrated high patient satisfaction along with improved abdominal contour.

Case 2

**Figure 4:**
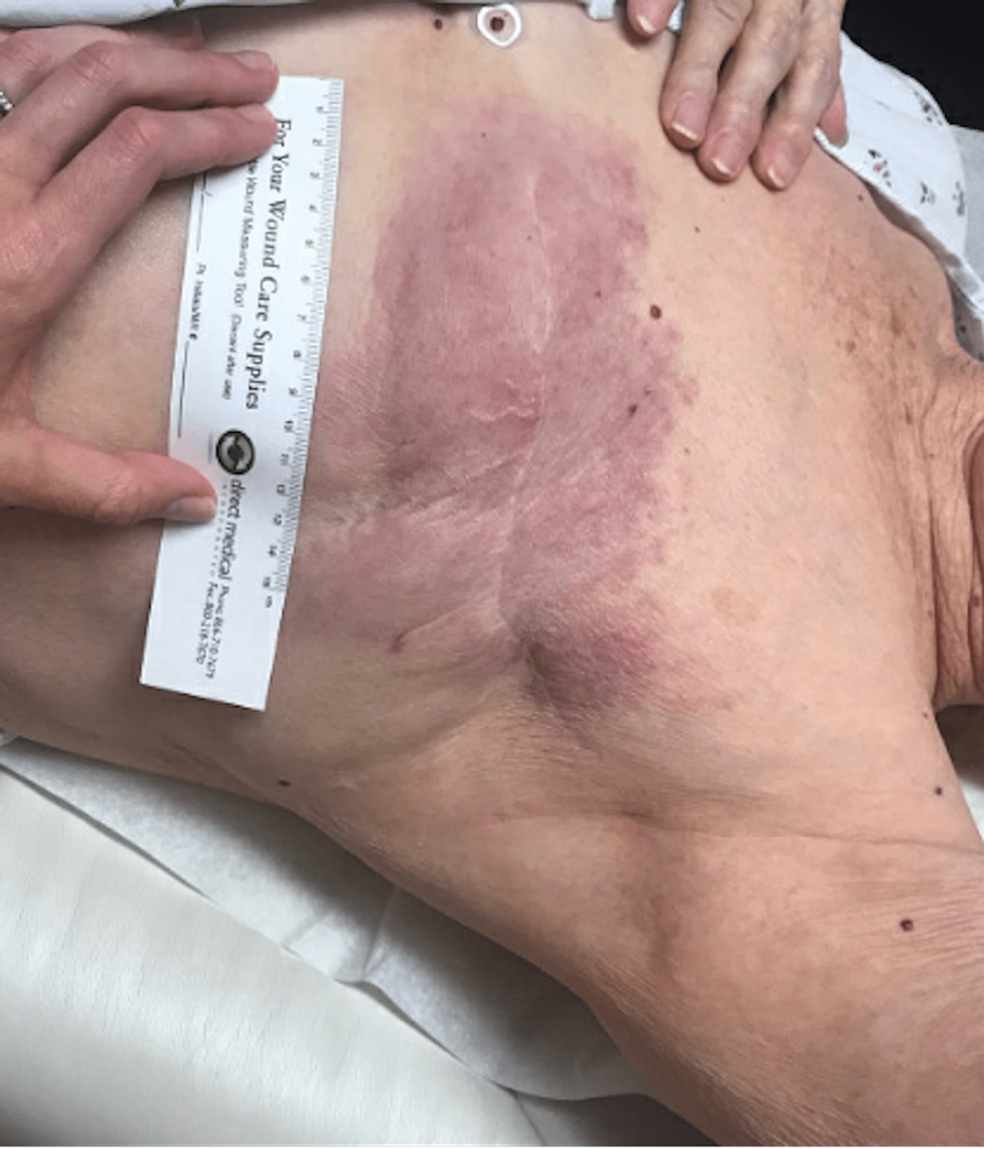
Case 2 - Initial presentation Recurrent left triple-negative invasive ductal carcinoma (21 x 12 cm) following neoadjuvant chemotherapy and a left modified radical mastectomy nine months prior to this presentation.

An 83-year-old female presented with a large area of locally recurrent triple-negative invasive ductal carcinoma (Figure 4). She had undergone a left modified radical mastectomy and neoadjuvant chemotherapy, nine months before this presentation. In addition to these complications, she had also had an ipsilateral thoracotomy in 2002 for the resection of a carcinoid tumor of the left lung. The final pathology report from her initial mastectomy revealed a 7 cm mass with a grossly positive superior margin and 4/14 positive lymph nodes. Unfortunately, the patient was not sent for re-excision or radiation following these reports. At the time of presentation, there was no evidence of distant disease.

Thus, it was decided to perform a re-excision to cure the recurrence of the disease. Given the prior history of thoracotomy, a rotational flap from the left upper abdomen or left latissimus dorsi were ruled out as potential flaps for coverage and a reverse abdominoplasty was elected.

**Figure 5:**
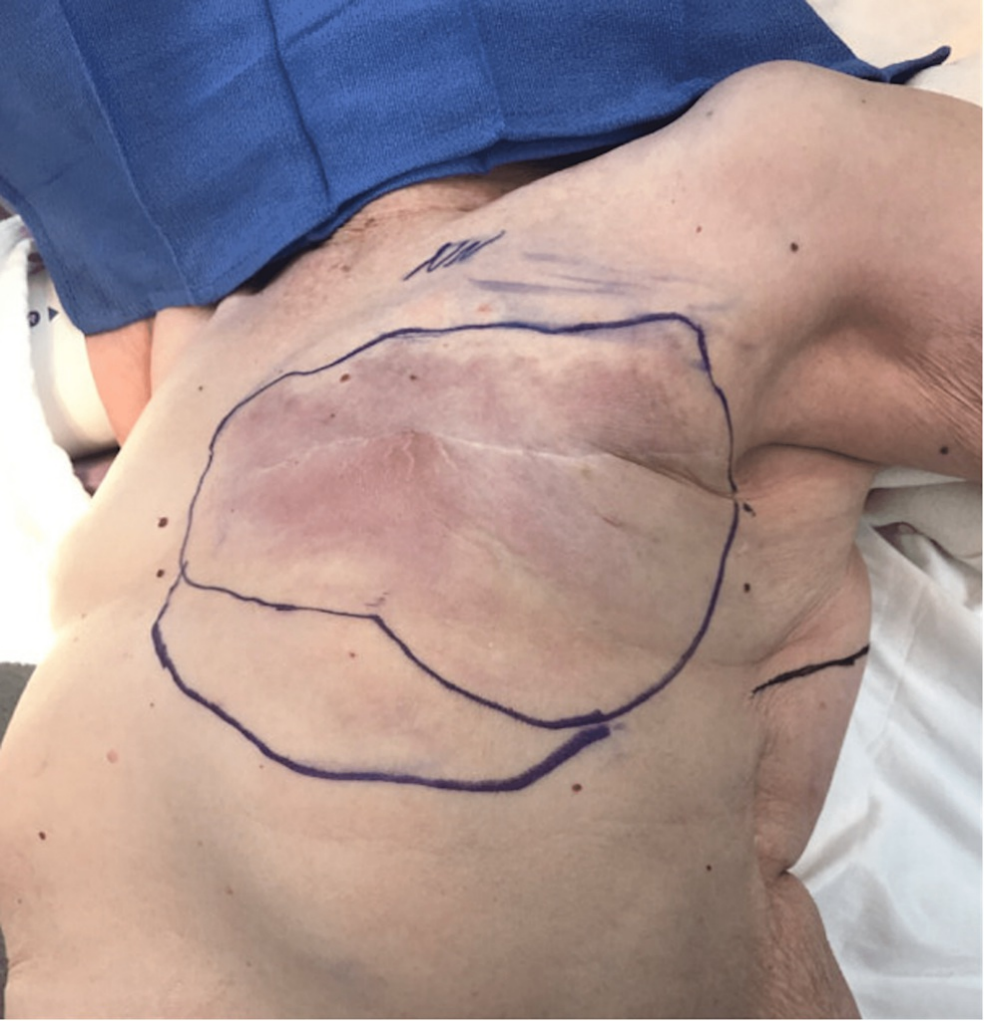
Case 2 - Preoperative markings Preoperative markings show the area (37 x 21 x 3 cm) that was excised. The area along the lower portion of the circle was also included as it was suspicious for involvement. The line along the lateral chest shows the prior thoracotomy scar.

The area of excision measured 37 x 21 x 3 cm (Figure 5). A portion of the left pectoralis muscle was also excised as her disease appeared to involve the muscle. There was no tension on closure. Final pathology revealed a triple-negative invasive ductal carcinoma with lymphovascular invasion and invasion into the pectoralis muscle deep resection margin.

**Figure 6:**
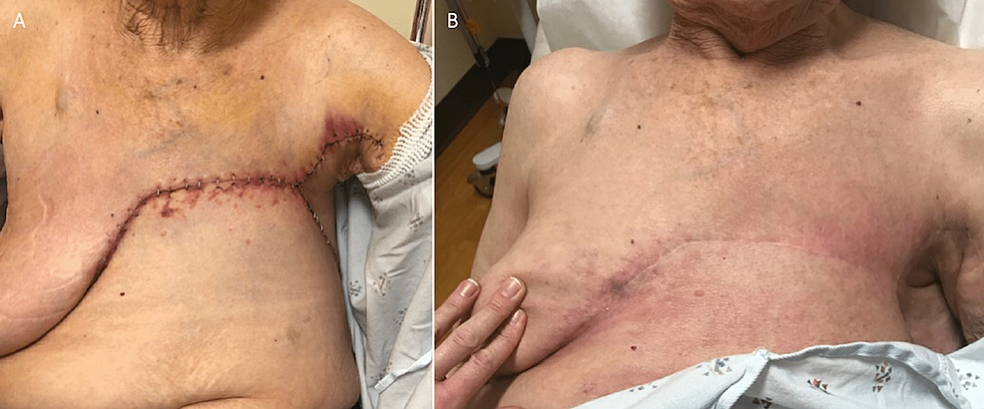
Case 2 - Follow-up (A) One-week follow-up postoperatively. (B) Six months follow-up post-operatively with a well-healed scar.

Postoperatively, the patient did not have any immediate complications (Figure 6). She received adjuvant radiation but declined further systemic therapy. Furthermore, six months postoperatively, skin changes were noted over the right breast, which was biopsied and revealed a triple-negative invasive ductal carcinoma (Figure 6). At that time, she was also noted to have developed a systemic disease.

Surgical technique

Preoperatively, the approximate extent of the disease was estimated and an assessment of abdominal skin and subcutaneous laxity was performed. Intra-operatively, the total extent of obvious disease was resected with wide clinical margins and clear operative margins were confirmed with final pathology.

Following the resection of the disease, undermining was performed inferiorly in the suprafascial plane along the inferior chest and upper abdomen as well as superior to the defect along the anterior chest up to the border of the clavicle. Undermining continued down to the level of the periumbilical perforators inferiorly, which were preserved. The chest incision was extended as a back cut with an inframammary incision under the right breast to allow for advancement. Undermining was performed along the right inferior chest wall and superior abdomen to allow mobilization across the defect. Two suction drains were placed deep in the advanced flap. Plication sutures were placed to anchor abdominal soft tissue to the chest wall and decrease dead space. The rest of the tissue was reapproximated with a suture without tension. The SPY System was used to evaluate flap perfusion.

## Discussion

There are generally four indications for chest wall reconstruction: the resection of a tumor, radiation injury, trauma, and infection. For defects of the chest wall limited to the soft tissue, as in our case, a thick soft tissue flap can provide enough stability [6]. There are numerous reconstruction options available for chest wall defects, including the pectoralis major muscle flap, the omental flap, the latissimus dorsi flap, the transverse rectus abdominis muscle (TRAM) flap, the deep inferior epigastric perforator (DIEP) free flap, the pedicled fasciocutaneous flap, and skin grafts [6].

The use of reverse abdominoplasty as a standalone reconstructive modality for chest wall soft tissue defects is a unique yet useful technique given the right situation [7,8]. It provides a highly vascularized tissue that is versatile and safe for reconstructive purposes in patients who do not want breast reconstruction and have abdominal soft-tissue laxity, and it provides adequate skin texture and color matching without significant surgical or donor site morbidity compared to other reconstructive options [9,10].

The reverse abdominoplasty flap also has the benefit of allowing for potential re-advancement for diseases with high-local recurrence risks, as noted in one case report [2]. However, scarring should be considered and if there is insufficient tissue, reverse abdominoplasty can be combined with other flaps [2].

It is well known that there is an increased risk of hematoma (31.5%), infection (27.2%), and deep venous or other thromboembolic complications (20.2%) following a traditional abdominoplasty [11]. However, there is little reported in the literature on complication rates following a reverse abdominoplasty. Both our patients had no immediate postoperative complications such as venous thromboembolism, hematoma, or infection.

Things that may preclude the use of a reverse abdominoplasty for reconstruction could be previous abdominal surgeries that may impede the vascularity of the flap. Reconstructive options, such as a DIEP or TRAM flap, may also be ruled out due to prior abdominal surgery. However, several studies exist describing the success of both DIEP and TRAM flaps for breast reconstruction following a previous laparotomy, traditional abdominoplasty, or numerous liposuction procedures [12-15]. Studies have also indicated the feasibility of abdominoplasty following DIEP harvesting. However, little literature exists regarding DIEP or TRAM following reverse abdominoplasty for breast disease resection in particular [16].

Given the subjectively good cosmetic results from the reverse abdominoplasty flaps in these patients and minimal to no donor site morbidity, we would consider this technique as a preferable reconstruction option for extensive Paget’s, large local recurrences, or other large defects involving the soft tissue of the anterior chest. Furthermore, the reverse abdominoplasty flap has been described following a case of bilateral breast disease as well [3]. Although not described in the literature and not desired by our patients in this series, breast reconstruction, either immediate or delayed, could follow a reverse abdominoplasty.

## Conclusions

In cases with extensive unilateral disease of the breast, the reverse abdominoplasty flap is a viable option for anterior chest wall soft tissue reconstruction. This technique offers a robust and safe reconstruction while providing ample soft tissue coverage of the defect. In settings of a limited plastic surgery department, reverse abdominoplasty is a surgical procedure that general surgeons or breast oncoplastic surgeons can keep in their toolkit when needing quick and extensive coverage of a soft tissue defect.
